# A PHMB-Functionalized Fully Absorbable Synthetic Matrix as a Novel Alternative to Biologics: Balancing Antibacterial Efficacy, Tissue Repair, and Safety

**DOI:** 10.3390/bioengineering13030353

**Published:** 2026-03-18

**Authors:** Sean Chen, Christopher Bibbo, John Starinski, Xianghua Xu, Chenhong Wang

**Affiliations:** 1 Kreate Medical Inc., 2300 Lakeview Parkway, Suite 700, Alpharetta, GA 30009, USA; johns@iuhs.edu (J.S.); xuxianghua@kreatemed.cn (X.X.); wangchenhong@kreatemed.cn (C.W.); 2 Foot & Ankle Surgery, Plastic Reconstructive & Microsurgery, Orthopaedic Trauma and MSK Infection Services, Rubin Institute for Advanced Orthopedics, Sinai Hospital of Baltimore, 2401 West Belvedere Avenue, Baltimore, MD 21215, USA; drchrisbibbo@gmail.com

**Keywords:** composite fiber, polyhexamethylene biguanide (PHMB), absorbable synthetic matrix, antibacterial wound matrix, soft tissue repair

## Abstract

**Highlights:**

**What are the main findings?**

• A fully absorbable PHMB-functionalized synthetic matrix mimicking extracellular matrix (ECM) architecture enables synchronized biphasic release and degradation, ensuring sustained antibacterial efficacy throughout wound healing while facilitating tissue repair.
• AATCC 100 assessments confirmed that the matrix maintains robust broad-spectrum bactericidal potency (log_10_ reduction values (LRV) > 4.0; >99.99% reduction) against six clinically relevant pathogens after 15 months of real-time aging, demonstrating superior stability for clinical translation.

**What are the implications of the main findings?**

• The PHMB Matrix demonstrates significantly accelerated healing compared to Xenograft AM in porcine full-thickness wound models by Day 22, with improved wound surface quality and histologically confirmed tissue remodeling and biocompatibility.
• The PHMB Matrix demonstrates sustained wound-site protection via a localized “enrichment effect” while maintaining a robust systemic safety profile through rapid, biphasic elimination.

**Abstract:**

Effective management of acute, complex, and chronic wounds requires constructs that simultaneously support tissue repair and provide sustained infection control. Biologic-derived materials, despite their regenerative potential, are limited by insufficient long-term antibacterial activity and susceptibility to enzymatic degradation. To overcome these limitations, a fully absorbable synthetic matrix composed of electrospun composite fibers functionalized with polyhexamethylene biguanide (PHMB) (hereafter, PHMB Matrix) was developed to mimic extracellular matrix architecture while enabling durable antibacterial performance. Quantitative assessment per AATCC 100 demonstrated robust broad-spectrum efficacy (>99.99% reduction) against six clinically relevant Gram-positive and Gram-negative pathogens, with potency retained after 15 months of real-time aging. The matrix’s interconnected fibrous architecture enables a controlled, biphasic PHMB release coordinated with biodegradation, sustaining antibacterial protection throughout a 28-day healing period. In porcine full-thickness wound models, the PHMB Matrix achieved 63.53% ± 12.0% wound area reduction by Day 22, demonstrating accelerated mid-phase healing compared to an antibacterial collagen control (*p* < 0.05 on Day 22), with both treatments achieving comparable near-complete closure by Day 28. Pharmacokinetic analysis confirmed localized drug enrichment with negligible systemic exposure. These findings establish the PHMB-functionalized synthetic matrix as a safe, effective, fully absorbable alternative to biologic-derived materials for soft tissue repair, offering sustained antibacterial efficacy and a favorable safety profile.

## 1. Introduction

The clinical management of complex cutaneous defects—encompassing chronic ulcers, acute traumatic wounds, and severe burns—is primarily challenged by the need to support tissue repair within contaminated environments [1,2,3,4]. While biologic-derived matrices, such as antibacterial collagen xenografts, are widely used in clinical practice, their utility is often constrained by unpredictable degradation kinetics in protease-rich wound environments [5,6]. Furthermore, many antibacterial-loaded matrices rely on surface-coating technologies for agent delivery, which frequently result in rapid depletion and a limited therapeutic window [7,8,9]. Accordingly, next-generation platforms must combine absorbability and structural stability with a precision-tuned antibacterial delivery system, ensuring that the therapeutic intervention aligns with the dynamic physiological microenvironment of the healing wound [1,10,11,12,13,14,15,16].

Polyhexamethylene biguanide (PHMB) exerts bactericidal effects through membrane disruption via electrostatic interaction between cationic biguanide groups and anionic phospholipids in bacterial membranes, resulting in cytoplasmic leakage and cell death within 5–10 min of contact [17,18,19,20]. To date, clinically significant resistance to PHMB has not been documented in wound-care settings under standard-of-care concentrations after decades of clinical use [21,22]. The minimum inhibitory concentrations (MICs) of PHMB against key wound and surgical-site pathogens have been characterized across multiple studies and are summarized here for reference: *S. aureus*, 0.5–2 µg/mL; *E. coli*, 0.5–2 µg/mL; *Pseudomonas aeruginosa*, 2–8 µg/mL; *MRSA*, approximately 2 µg/mL [23,24]. In protein-laden environments or wound-exudate-simulating media, effective inhibitory concentrations may be several-fold higher than standard broth MIC values, reflecting the binding of cationic PHMB to serum proteins and wound-bed components [1,25]. From a design standpoint, absorbable antibacterial constructs should achieve temporal synchronization between PHMB release kinetics and matrix biodegradation, such that local drug concentrations remain above the MIC of target pathogens throughout the infection-susceptible phases of wound healing [26,27]. The acute wound-healing cascade comprises four overlapping phases: hemostasis (minutes to hours), inflammation (days 1–4), proliferation (days 4–21), and remodeling (beginning around Day 21 and continuing for up to 1–2 years) [24,28]. The primary window of infection vulnerability—and therefore the minimum duration of antibacterial coverage—spans approximately the first 21 days post-injury, encompassing the inflammatory and proliferative phases during which the wound bed lacks an intact epithelial barrier [27,29,30].

Although PHMB has been incorporated into various electrospun platforms—including PLA [31], cellulose acetate/polyester urethane [32], PCL/PVA [33], and silk fibroin matrices [34]—these systems typically exhibit poorly controlled release kinetics (e.g., predominant burst release within 1–4 h), employ non-degradable matrices, or lack in vivo pharmacokinetic validation; to date, none have simultaneously achieved biphasic release synchronized with complete scaffold biodegradation.

Herein, we report a fully absorbable composite electrospun matrix comprising poly (lactic-co-glycolic acid) (PLGA) and polydioxanone (PDO) fibers, functionalized with polyhexamethylene biguanide (PHMB) as the active antimicrobial agent and Poloxamer 188 (P188) as a hydrophilic surface modifier (hereafter referred to as PHMB Matrix). Within this design, each component serves a distinct role: PHMB confers broad-spectrum bactericidal activity at the wound interface; P188 enhances surface wettability and conformability to irregular wound topography; and the PLGA/PDO dual-fiber scaffold functions as a diffusion-barrier carrier governing biphasic PHMB release kinetics, while simultaneously serving as a degradable template mimicking the extracellular matrix (ECM), conducive to guided tissue repair. Notably, hydrolytic degradation of the PLGA/PDO scaffold yields lactic and glycolic acids, which locally acidify the wound microenvironment—a condition demonstrated to promote angiogenesis [35] and potentiate innate antimicrobial defense [36]. We hypothesized that the dual-fiber architecture would enable biphasic PHMB release—initial burst for acute infection control, followed by sustained diffusion—synchronized with matrix degradation [5]. We validated this hypothesis through: (1) in vitro characterization of antibacterial efficacy, release kinetics, and degradation; (2) in vivo assessment of efficacy and biocompatibility in porcine wound models; and (3) pharmacokinetic studies in rats to establish safety margins.

The matrix demonstrated durable antibacterial activity, maintaining high log_10_ reduction values (LRV) even after extended storage. The therapeutic performance of the PHMB Matrix was further validated in porcine models [37,38,39]. In a full-thickness defect model, the PHMB Matrix significantly accelerated re-epithelialization by Day 22 compared with an antibacterial collagen control (63.53% ± 12.0% vs. 49.47% ± 9.0%, *p* < 0.05). In parallel, systemic safety was assessed via a rodent pharmacokinetic model to quantify the disposition of PHMB under repeated dosing conditions [17,18]. PHMB is preferentially retained at the wound site and surrounding tissues, with negligible systemic uptake. By bridging the material-driven degradation–repair balance with quantified preclinical data, this study establishes the PHMB Matrix as a fully absorbable synthetic alternative that successfully balances antibacterial efficiency, tissue repair, and safety—addressing the inherent limitations of biologic-derived materials.

## 2. Experimental Section

### 2.1. Preparation, Characterization, and In Vitro Performance of the PHMB Matrix

#### 2.1.1. Preparation and Characterization of the PHMB Matrix

PLGA and PDO were of medical grade for wound-contact use; PHMB and P188 were of pharmaceutical grade, meeting applicable pharmacopoeial specifications. The spinning dopes were prepared by dissolving PLGA, PDO, or their combination in an organic solvent system to reach an optimized total polymer concentration of 8–15% (*w*/*v*); PHMB was incorporated into one solution and P188 into the other to ensure balanced hydrophilicity.

The antibacterial PHMB Matrix was fabricated via co-electrospinning using a TL Series Electrospinning System (Tongli Tech Co., Ltd., Shenzhen, China) to produce an integrated composite comprising two distinct fiber types [40]. Both fibers are composites: the first is functionalized with PHMB, and the second is incorporated with P188. The matrix achieves a nominal PHMB concentration of 25 mg/g relative to the total polymer mass to provide sustained antibacterial activity.

The electrospinning process was conducted at room temperature (chamber humidity 30–50%), with a solution flow rate of 5–15 mL/h, an applied voltage of 15–25 kV, and a tip-to-collector distance of 10–20 cm. The resulting non-woven fibrous matrix was thoroughly dried to ensure complete solvent removal. The thickness and porosity of the PHMB Matrix can be tailored by adjusting electrospinning parameters. All samples were sterilized via Gamma irradiation prior to experimental use.

The porosity (P) was determined via the ethanol displacement method (*n* = 5). Samples were dried at 37 °C for 2 h to obtain the dry mass (*m*). A 25 mL volumetric flask was filled with anhydrous ethanol to the mark (*m*_1_). After immersing the sample for 30 min, excess ethanol above the mark was removed, and the total mass was recorded as *m*_2_. The sample was then removed to measure the residual mass (m_3_). P was calculated as:

P = (*m*_2_
*− m*_3_ − *m*)/(*m*_1_ − *m*_3_)

The thickness of the matrix was measured using a digital thickness gauge with a precision of 0.001 mm. The absorption capacity was evaluated via the non-expansion absorption method in accordance with YY/T 0471.1-2004 [41], wherein samples were immersed for 30 min, drained, and blotted to remove surface moisture prior to gravimetric analysis. The tensile properties were characterized using an electronic universal testing machine (Model FYW-L, Jinan Fangyuan Test Instrument Co., Ltd., Jinan, China) at a tensile rate of 200 mm/min.

The morphology of the PHMB Matrix was observed using a scanning electron microscope (SEM, ZEISS, Oberkochen, Germany). Prior to imaging, the samples were sputter-coated with a thin layer of gold under vacuum to enhance electrical conductivity. Fiber diameters were measured using ImageJ software (version 1.54; National Institutes of Health, Bethesda, MD, USA), with at least 100 fibers measured per condition; diameter distributions were presented as histograms (bin size = 0.2 μm).

#### 2.1.2. In Vitro Release Kinetics of PHMB

The release profile was evaluated according to the Chinese Pharmacopoeia 2020 (Method 3, Small Cup Method). Matrix samples (2.5 × 2.5 cm^2^) were immersed in 100 mL of purified water or 0.05 M NaOH at 37 ± 0.5 °C with a stirring speed of 200 rpm (pH ≈ 12.7). At predetermined intervals (water: 1 h, 2 h, 4 h, 12 h, 24 h, 72 h, 168 h; NaOH: 1 h, 2 h, 4 h, 24 h, 48 h), 10 mL of the medium was withdrawn and replaced with fresh solvent. PHMB concentrations were quantified via UV-Vis spectrophotometry at 235 nm against a linear calibration curve prepared from PHMB standard solutions (0.5–15 mg/L). All measurements were performed with six replicates (*n* = 6).

#### 2.1.3. Quantitative Antibacterial Efficacy and Stability

The antibacterial potency of the PHMB Matrix was evaluated against six clinically relevant pathogens using a modified American Association of Textile Chemists and Colorists (AATCC) Test Method 100-2019 [42]. To rigorously validate the functional stability of the device throughout its intended lifecycle, samples subjected to 15 months of real-time aging were utilized (25 °C ± 2 °C, RH 60% ± 5%). To further simulate a “worst-case” clinical scenario representing the maximum 7-day in-use wear time, these aged samples underwent a 7-day pre-conditioning step in simulated wound fluid (SWF). The SWF was formulated according to Svensby et al. and comprised the following components: 110 mM NaCl, 2.2 mM CaCl_2_, 2.7 mM KCl, 0.5 mM MgCl_2_, 34 g/L bovine serum albumin, 1.3 mM KH_2_PO_4_, and 20 mM NaHCO_3_ [43]. A placebo matrix without PHMB served as the control. Three independent lots were tested with two replicates per condition. This pre-depletion and organic soiling process ensures that the antibacterial performance is assessed at the device’s limit of practical application, reflecting its sustained efficacy within a bio-relevant microenvironment.

Bactericidal activity was quantified against the following six American Type Culture Collection (ATCC) reference strains: *Staphylococcus aureus* (*S. aureus*, ATCC 6538), *Bacillus subtilis* (*B. subtilis*, ATCC 6633), *Staphylococcus epidermidis* (*S. epidermidis*, ATCC 12228), *Escherichia coli* (*E. coli*, ATCC 25922), *Pseudomonas aeruginosa* (*P. aeruginosa*, ATCC 9027), and *Klebsiella pneumoniae* (*K. pneumoniae*, ATCC 4352).

Prior to the assay, the bacteria were cultured in Tryptic Soy Broth (TSB) at 37 °C for 24 h to reach the mid-logarithmic growth phase. The suspension was subsequently adjusted to a concentration of 10^6^ colony-forming units (CFU)/mL using sterile saline.

Specimens (five swatches per replicate) were pre-conditioned in simulated wound fluid at 37 ± 2 °C for 168 h to simulate 7-day clinical wear, then inoculated with 1 mL bacterial suspension (1–3 × 10^6^ CFU/mL). Bacterial counts were determined at 0 h and 24 h contact times. After incubation, specimens were neutralized in 100 mL Dey/Engley (D/E) broth, serially diluted, membrane-filtered (0.45 μm), plated on tryptic soy agar, and incubated at 35 ± 2 °C for 24 h. Neutralization validity (survival rate greater than 70%) and recovery efficiency were confirmed prior to efficacy calculation. Uninoculated controls verified the absence of contamination. The Log Reduction Value (LRV) and reduction percentage are calculated using the following formulas:

LRV= lg C − lg A,

Reduction percentage = (C − A)/C × 100%

where A is the number of microorganisms recovered from the inoculated test articles at 24 h contact time, C is the number of microorganisms recovered from the inoculated test articles immediately after inoculation (at 0 contact time).

Acceptance criteria: >4 log reduction, i.e., >99.99% reduction is required to demonstrate effective antibacterial performance.

#### 2.1.4. In Vitro Biodegradation and pH Monitoring

In Vitro degradation profile of the matrix in physiological saline was evaluated under multiple conditions. Hydrolytic degradation in phosphate-buffered saline (PBS: 8.0 g/L NaCl, 0.2 g/L KCl, 3.76 g/L Na_2_HPO_4_·2H_2_O, 0.2 g/L KH_2_PO_4_, pH 7.4) at 37 °C was conducted in accordance with ISO 10993-13 [44] and ASTM F1635-16 [45] standards. Samples (5 × 7.5 cm^2^) were immersed in 40 mL PBS, with complete solution replacement every 7 days over 10 weeks (*n* = 3 per time point). Degradation in non-buffered physiological saline (PSS: 9 g/L NaCl) was investigated in parallel, as the addition of acid or alkaline agents may accelerate hydrolysis of PLGA and PDO. Samples (5 × 5 cm^2^) were immersed in 40 mL PSS at 37 °C and 45 °C, respectively, each over 10 weeks, with complete solution replacement every 7 days (*n* = 3 per time point). To simulate the enzymatic environment of wound exudate, samples (1.25 × 1.88 cm^2^) were further immersed in 20 mL PBS supplemented with 4 g/L lipase at 45 °C, with complete solution replacement every 2 days over 28 days (*n* = 3 per time point) [46]. At predetermined intervals, samples were retrieved, rinsed with distilled water, and dried to a constant weight in a vacuum oven.

The weight loss percentage was calculated using the formula

Weight loss (%) = (*W*_0_ − *W_t_*)/*W*_0_ × 100%

where *W*_0_ and *W_t_* represent the dry weight of samples on Day 0 and Day *t*, respectively. All measurements were performed in triplicate to ensure statistical reproducibility.

Simultaneously, the pH of the medium was monitored to evaluate the influence of degradation byproducts on the buffering medium. Post-degradation fiber morphology and diameters were characterized by SEM and ImageJ (version 1.54) as described in Section 2.1.1.

### 2.2. Safety and Effectiveness Evaluation of PHMB Matrix in Porcine Full-Thickness Defects

The clinical translation potential and safety of the PHMB Matrix were rigorously validated using a Bama miniature pig full-thickness defect model. Four minipigs were used in this study, housed and cared for under standard laboratory conditions in accordance with animal welfare guidelines. All experimental protocols were approved by the Institutional Animal Care and Use Committee (IACUC) (see Section 2.5 for details). At the end of the study, animals were humanely euthanized for tissue collection following established veterinary welfare protocols.

A total of 40 symmetrical full-thickness excisional wounds (3 cm in diameter, covered with 3.75 × 3.75 cm^2^ matrices) were established on the dorsal region of the animals (10 defects per pig, 5 on each side). Wounds were randomly assigned to receive either the PHMB Matrix or the Antibacterial Cross-linked Xenograft (Xenograft AM, control). The matrices were secured with sterile gauze and replaced every seven days following saline irrigation to minimize tissue trauma.

Wound healing was monitored through digital photography and planimetric analysis (ImageJ) on days 0, 7, 15, 22, and 28. Healing efficacy was quantified by wound area reduction rates.

Safety was assessed via continuous clinical observation and histological evaluation of the wound sites. Upon scheduled necropsy on Day 15 and Day 28, gross tissue observations and histopathological examinations were performed to evaluate tissue regeneration and the local inflammatory response.

Wound tissues and major organs were fixed in 10% neutral buffered formalin, paraffin-embedded, and stained with Hematoxylin and Eosin (H&E) and Masson’s Trichrome. The local biological response and biocompatibility were evaluated in strict accordance with ISO 10993-6:2016 [47] using a semi-quantitative scoring system (0–4 scale), categorized into Cellular Response (*I*) and Tissue Response (*TR*). Cellular Response assessed polymorphonuclear cells, lymphocytes, plasma cells, macrophages, and giant cells, quantified by the number of cells per high-power field (400×): 0 (none), 1 (minimal, 1–5 cells), 2 (mild, 6–10 cells), 3 (moderate, 11–20 cells), and 4 (severe, >20 cells or packed). Tissue Response evaluated neovascularization and fibrosis/matrix formation. Neovascularization was scored as: 0 (none), 1 (minimal, <3 capillaries with fibroplasia), 2 (mild, 4–7 capillaries), 3 (moderate, 8–12 capillaries), and 4 (extensive, >12 capillaries per field). Fibrosis was graded as: 0 (none), 1 (minimal collagen layer), 2 (mild, thin band), 3 (moderate, thick band), and 4 (extensive/mature, dense collagen bundles filling the defect).

In accordance with the 3R principles (Replacement, Reduction, and Refinement), the study design integrated comprehensive histopathological grading with continuous clinical health monitoring as the definitive terminal endpoint for safety evaluation. The absence of severe localized toxicity (Grade 4), combined with stable clinical growth kinetics and preserved organ integrity confirmed at gross necropsy, provided a robust safety profile that eliminated the necessity for invasive systemic hematological monitoring, thereby minimizing animal distress while maintaining scientific rigor.

### 2.3. Pharmacokinetic (PK) Study in SD Rats

To evaluate systemic safety, absorption and distribution of PHMB were studied in specific-pathogen-free (SPF)-grade Sprague–Dawley (SD) rats (*n* = 8 for pharmacokinetics, 4 males and 4 females; *n* = 6 per time point for local tissue retention, 3 males and 3 females). Prior to each PHMB Matrix application, animals were fasted for at least 12 h with ad libitum access to water; food was restored 3 h post-application. Full-thickness circular wounds (3 cm diameter, covered with 3.75 × 3.75 cm^2^ PHMB Matrix) were created on the dorsal skin, and PHMB Matrix patches (25 mg/g, theoretical PHMB dose 1525 µg per patch) were applied directly onto the wound bed and secured with gauze bandage, weekly on days 0, 7, 14, and 21. Blood samples (0.3 mL) were collected via the orbital venous plexus at predefined intervals from 2 h up to 56 d; plasma was separated by centrifugation (4 °C, 12,000 rpm, 5 min) within 60 min and stored at −70 °C [48]. For local tissue retention, separate cohorts were euthanized by abdominal aortic exsanguination on days 22, 24, 28, 35, and 56; wound-site skin tissue (i.e., the tissue at and surrounding the wound bed) was excised, homogenized (1 g:10 mL), and stored at −70 °C until analysis. Plasma and skin homogenate PHMB concentrations were quantified using a validated liquid chromatography-tandem mass spectrometry (LC-MS/MS) method (ExionLC system coupled with an AB SCIEX Triple Quad 6500 mass spectrometer; AB Sciex LLC, Framingham, MA, USA) [49]. Analytes were separated on a CAPCELL PAK ADME HR column (100 × 4.6 mm, 5 μm; 45 °C) using gradient elution with methanol and 0.1% formic acid–10 mM ammonium formate–acetonitrile at 0.5 mL/min. The mass spectrometer was operated in positive ESI mode with multiple reaction monitoring (MRM) transitions of *m*/*z* 367.3 → 184.2 for PHMB and *m*/*z* 256.2 → 167.1 for the internal standard (diphenhydramine). The lower limit of quantification (LLOQ) was established at 10 ng/mL, with accuracy and precision within ±20% per ICH M10 bioanalytical method validation guidelines [50]. Samples with no detectable signal were reported as below the limit of detection (<LOD).

### 2.4. Statistical Analysis

All quantitative data were expressed as mean ± SD, except pharmacokinetic data (mean ± SEM). Normality of wound healing rates was assessed; normally distributed data were compared by two-sample *t*-test, and non-normally distributed data by Wilcoxon rank-sum test (*p* ≤ 0.05 or *p* ≤ 0.01). Antibacterial sample size adequacy was confirmed by prospective power analysis (Minitab LLC, State College, PA, USA; α = 0.05, two-sided; power ≥ 0.90), with acceptance set at LRV > 4 (>99.99% reduction). Biocompatibility was scored per ISO 10993-6:2016: Total = (*I* × 2) + *TR*; a relative score of 0.0–2.9 was classified as “no or minimal reaction.” Fiber diameters and wound areas were measured using ImageJ (version 1.54).

### 2.5. Quality Assurance

To minimize potential bias inherent to manufacturer-sponsored research, all preclinical animal studies were conducted by independent Contract Research Organizations under full Good Laboratory Practice (GLP) compliance: the porcine wound-healing study at WuXi AppTec (Suzhou, China), an AAALAC-accredited facility, and the rat pharmacokinetic study at Tianjin Tiancheng Biotechnology (Tianjin, China). Wound site assignments in the porcine study were randomized using a Latin square design, ensuring that each animal received no more than one wound per treatment group. All study protocols were finalized and IACUC-approved prior to study initiation (protocol L27-0002-SGC for the porcine study). Wound planimetric analyses were conducted by personnel blinded to treatment allocation, and histopathological scoring was performed by a qualified lead pathologist in accordance with GLP-defined procedures. All raw data are archived per GLP requirements and remain available for regulatory or peer-review inspection upon reasonable request.

## 3. Results

### 3.1. Characterization of the PHMB Matrix

The matrix was fabricated via co-electrospinning to produce an integrated matrix comprising two distinct fibers with specialized functionality. Each fiber is made of composite material with different ratios of PLGA and PDO [40,51]. This dual-composite architecture interleaves a hydrophilic component with a therapeutic carrier for antibacterial delivery [10,12]. By blending these functional fibers, the resulting matrix maintains a balance between structural integrity, fluid management, and controlled drug release.

#### 3.1.1. Structural, Morphological and Mechanical Characterization

**Figure 1:**
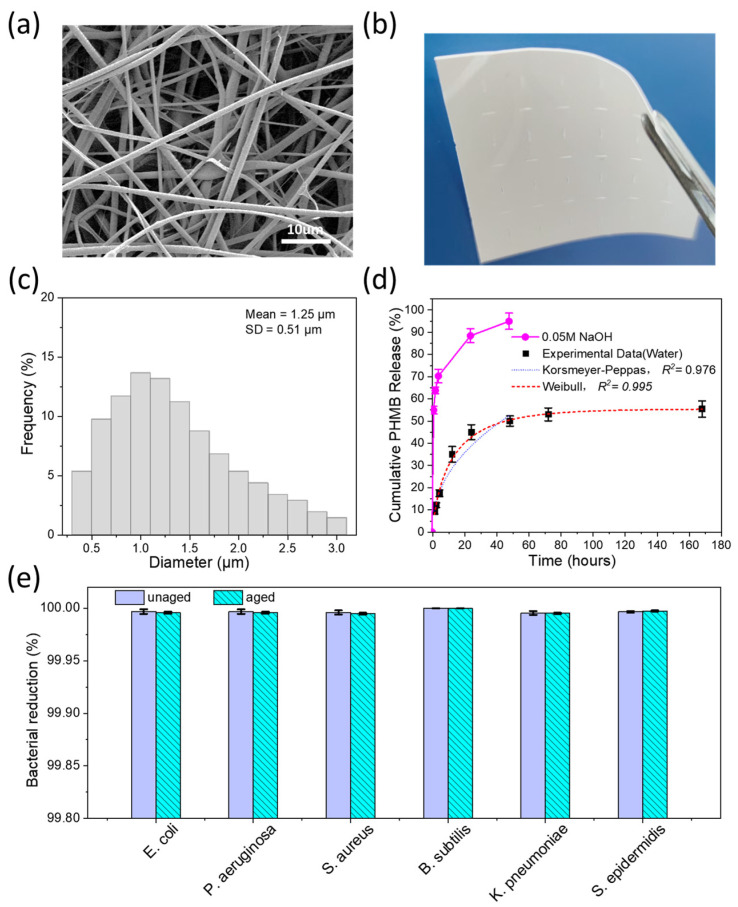
Morphological, physical, and functional characterization of the PHMB Matrix. (**a**) SEM image showing the fibrous microstructure of the PHMB Matrix (Scale bar: 10 μm). (**b**) Digital photograph of the flexible and macroscopic appearance of the matrix. (**c**) Fiber diameter distribution of the PHMB Matrix measured from SEM images using ImageJ (*n* = 200–300). Bin size = 0.2 μm. The average fiber diameter was 1.25 ± 0.51 μm. (**d**) Cumulative release profiles of PHMB from the matrix in 0.05 M NaOH solution and deionized water over 168 h and kinetic modeling of the aqueous release profile. Experimental data (water, black squares, mean ± SD) were fitted with the Korsmeyer-Peppas model (blue dotted line; *Q* = 9.59·*t*^0.44^, *R*^2^ = 0.976, applicable for *t* = 0.5–48 h) and the Weibull model (red dashed line; *Q* = 55.48 · [1 − exp(−(t/13.84)^0.71^)], *R*^2^ = 0.995, applicable for *t* = 0–168 h). The Korsmeyer-Peppas diffusional exponent *n* = 0.44 indicates a Fickian diffusion-dominated release mechanism. The Weibull plateau parameter (*Q_max_* = 55.48%) defines the upper boundary of diffusion-accessible PHMB. (**e**) Broad-spectrum Antibacterial Efficacy: Quantitative analysis per AATCC 100 demonstrates that the PHMB Matrix maintains broad-spectrum efficacy (*LRV* > 4.0, corresponding to >99.99% bacterial eradication) after 15-month aging. Potent bactericidal activity was confirmed against clinically relevant Gram-positive (*S. aureus*, *B. subtilis*, *S. epidermidis*) and Gram-negative (*E. coli*, *P. aeruginosa*, *K. pneumoniae*) pathogens.

The PHMB Matrix exhibits a highly porous, interconnected fibrous network as characterized by SEM (Figure 1a). Quantitative image analysis of SEM micrographs (*n* = 200–300 fibers) revealed a mean fiber diameter of 1.25 ± 0.51 µm with a unimodal distribution ranging from 0.5 to 3.0 µm (Figure 1c). Physical characterization demonstrated that the matrix possessed a thickness of 200 ± 50 µm, a porosity of 85 ± 5%, and an absorption capacity of approximately 400 ± 20% of its dry weight. This micron-scale fibrous architecture provides an expansive surface area designed to facilitate efficient drug loading and exudate management [52,53,54]. Macroscopically, the matrix demonstrates structural flexibility (Figure 1b), enabling non-invasive visual monitoring of the wound bed during treatment. The prepared matrix exhibited a tensile strength of ~0.5 MPa with a peak tensile load exceeding 4.2 N. These mechanical indices are comparable to those of commercial monolayered benchmarks (0.04–0.77 MPa) [55], underscoring the material’s capability to withstand the physical demands of wound management applications.

#### 3.1.2. In Vitro Drug Release Kinetics

The selection of purified water and 0.05 M NaOH (pH ≈ 12.7) as release media, rather than SWF, was based on three considerations: (i) BSA (34 g/L in SWF) exhibits broad peptide-bond absorption overlapping the PHMB detection wavelength (235 nm); (ii) polycationic PHMB undergoes electrostatic sequestration by anionic serum proteins [1,25], systematically underestimating true cumulative release; and (iii) the two-medium design decouples diffusion-controlled release (water, intact matrix) from erosion-controlled release (NaOH, hydrolyzed matrix), enabling biphasic profile quantification. Bio-relevance is independently validated by AATCC 100 testing after 7-day SWF pre-conditioning (Section 2.1.3), in vivo pharmacokinetic tissue data (Section 2.3), and porcine wound efficacy (Section 2.2).

The PHMB-functionalized matrix exhibits a biphasic release profile governed by its interconnected fibrous architecture (Figure 1d). The released PHMB concentration exceeds the Minimum Inhibitory Concentration (MIC) within 1 h via rapid surface desorption (~10% elution in neutral aqueous medium, corresponding to ~10.8 μg/cm^2^), establishing a prompt antibacterial barrier against acute infection [56,57]. The clinical relevance of this burst release was evaluated under a worst-case dilution scenario. *S. aureus*, including MRSA, is the most frequently isolated pathogen from infected wounds (MIC = 2 μg/mL) [24,58]. Even at the extreme exudate rate of 4.7 mL/cm^2^/day—the upper bound reported for full-thickness pressure ulcers [59]—this burst release yields an estimated local concentration of ~55 μg/mL, 27-fold above the MRSA MIC, yet still ~7-fold below that of commercial PHMB irrigation solutions (0.04%) and >360-fold below the contact sensitization threshold (2%) [24]. Moreover, since PHMB release from the PHMB Matrix is driven by fluid-mediated swelling and diffusion, reduced exudate inherently limits the released dose; where residual contact transfer does occur, the drug partitions into the underlying tissue volume rather than accumulating in a confined surface layer. The local concentration is therefore self-regulating across clinically relevant wound conditions, confirming a wide safety margin. Following the initial burst, the system transitions to a sustained diffusion-dominated phase in neutral media, reaching a stable plateau of 55.41% ± 3.68% by 168 h. The overall release kinetics were well fitted by both the Korsmeyer–Peppas (*R*^2^ = 0.976) and Weibull (*R*^2^ = 0.995) models, consistent with a diffusion-dominated transport mechanism throughout the 168-h release period. This sequestration within the hydrophobic polymeric core ensures a long-term therapeutic reservoir, maintaining efficacy throughout the critical proliferative phase of healing [60,61].

In contrast, accelerated alkaline hydrolysis (0.05 M NaOH) triggers a rapid, erosion-mediated elution, achieving 94.87% ± 3.67% release within 48 h as the fibrous matrix undergoes pseudo-enzymatic degradation [62,63]. This sharp divergence confirms that PHMB delivery is strictly synchronized with the matrix’s bio-resorption kinetics. By aligning drug elution with the three-stage biodegradation, this synthetic interface effectively eliminates the “sterile window gap” characteristic of surface coatings, providing comprehensive and durable antibacterial protection throughout the 28-day wound healing cycle.

#### 3.1.3. Broad-Spectrum Antibacterial Efficacy and Stability

The PHMB-functionalized matrix demonstrated potent broad-spectrum bactericidal activity against a panel of six clinically relevant Gram-positive (*S. aureus*, *B. subtilis*, *S. epidermidis*) and Gram-negative (*E. coli*, *P. aeruginosa*, *K. pneumoniae*) ATCC reference strains according to AATCC 100 protocols (Figure 1e) [19,43]. To assess performance at the device’s functional limit, specimens underwent 15 months of real-time aging (25 °C, 60% RH) followed by 7-day pre-conditioning in SWF at 37 °C, allowing the diffusion-accessible PHMB to reach its release plateau (~55%, Figure 1d) prior to bacterial challenge. Despite this substantial pre-depletion, the matrix achieved significant Log Reduction Values (LRV > 4.0, corresponding to >99.99% bacterial eradication) against all six tested strains, highlighting the matrix’s exceptional shelf-life and sustained antibacterial protection for chronic wound care [64].

#### 3.1.4. In Vitro Degradation and Microenvironmental Stability

**Figure 2:**
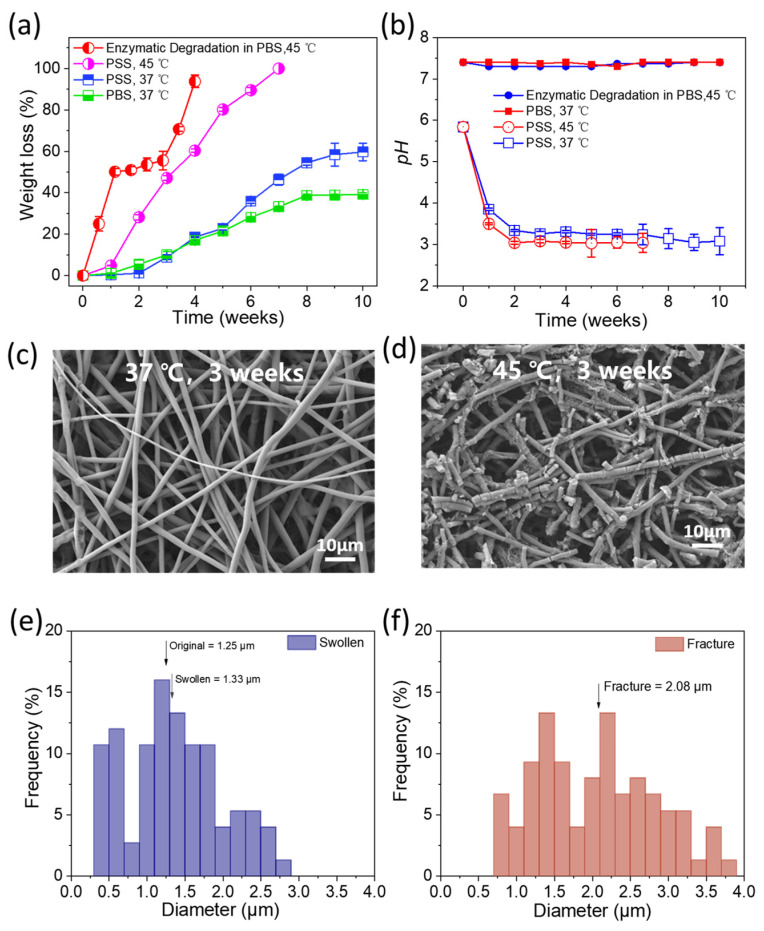
In vitro degradation behaviors and morphological characterization. (**a**) Cumulative weight loss of PHMB Matrix in phosphate-buffered saline (PBS) and physiological saline solution (PSS) at 37 °C and 45 °C over 10 weeks. The results demonstrate accelerated degradation at elevated temperatures and within the enzymatic environment (Enzymatic Degradation in PBS, 45 °C). (**b**) Variations in pH values of the surrounding media during the 10-week degradation period. A drastic decline in pH is observed in the PSS groups due to the accumulation of acidic by-products (lactic and glycolic acids) from the PLGA segments. In contrast, the PBS group maintains a stable physiological pH (7.3~7.4), demonstrating effective buffering capacity. (**c**) Representative SEM image of swollen fibers (37 °C, 3 weeks, PSS). (**d**) Representative SEM image of fibers at fracture stage (45 °C, 3 weeks, PSS). Scale bars = 10 μm. (**e**) Fiber diameter distribution of the swollen sample. The arrow indicates the original mean diameter (1.25 μm). The mean diameter of swollen fibers was 1.33 ± 0.58 μm. (**f**) Fiber diameter distribution of the fractured sample. The mean diameter increased to 2.08 ± 0.78 μm. Fiber diameters were measured from SEM images using ImageJ (approximately 100 fibers per condition).

The degradation profile of the PHMB Matrix was characterized under four conditions to decouple the contributions of hydrolysis, buffering capacity, temperature, and enzymatic activity (Figure 2a) [65]. In buffered PBS at 37 °C, the fibrous matrix maintained structural integrity with negligible mass loss (<2%) during the first two weeks, followed by progressive degradation reaching ~40% by Week 10. In non-buffered physiological saline (PSS) at 37 °C, degradation was moderately accelerated (~60% by Week 10) and further compressed at 45 °C, achieving near-complete mass loss by Week 10. The most rapid degradation occurred under enzymatic conditions (lipase/PBS, 45 °C), with near-complete mass loss within 4 weeks, confirming the synergistic contribution of enzymatic catalysis to polyester chain scission [46].

Concurrent pH monitoring revealed a medium-dependent divergence (Figure 2b): in non-buffered PSS, pH declined from ~5.8 to ~3.0–3.5 within the first week at both temperatures, while remaining stable at ~7.4 in buffered PBS throughout. The “pH–weight loss lag” suggests that hydrolytic scission of PLGA and PDO ester bonds generates soluble acidic products—lactic acid and glycolic acid from PLGA, glycolic acid from PDO—that diffuse into the surrounding medium even before significant macroscopic mass loss occurs [13]. Such local acidification may confer additional therapeutic benefit, as a mildly acidic wound milieu has been reported to favor certain healing phases, including antimicrobial defenses and re-epithelialization [36,66,67].

SEM and fiber diameter analysis corroborated the biphasic degradation process (Figure 2c–f). At 37 °C (3 weeks), fibers exhibited moderate swelling (mean diameter: 1.25 → 1.33 μm) while retaining continuous morphology (Figure 2c,e); at 45 °C (3 weeks), fibers underwent segmental fragmentation with a concomitant increase in mean diameter to 2.08 μm, yet retained their characteristic fibrous morphology (Figure 2d,f). Notably, under both conditions, the matrix preserved its interconnected porous architecture, thereby maintaining a structural template conducive to cell adhesion, migration, and extracellular matrix deposition during tissue repair. This progression from intact swelling to fragmentation aligns with the preceding release data: the preserved fibrous architecture during the initial weeks underpins the diffusion-controlled release plateau (~55% at 168 h, Figure 1d), while subsequent matrix erosion is expected to progressively liberate the remaining sequestered PHMB as a long-term therapeutic reservoir.

### 3.2. Efficacy and Biocompatibility in Porcine Wound Models

**Figure 3:**
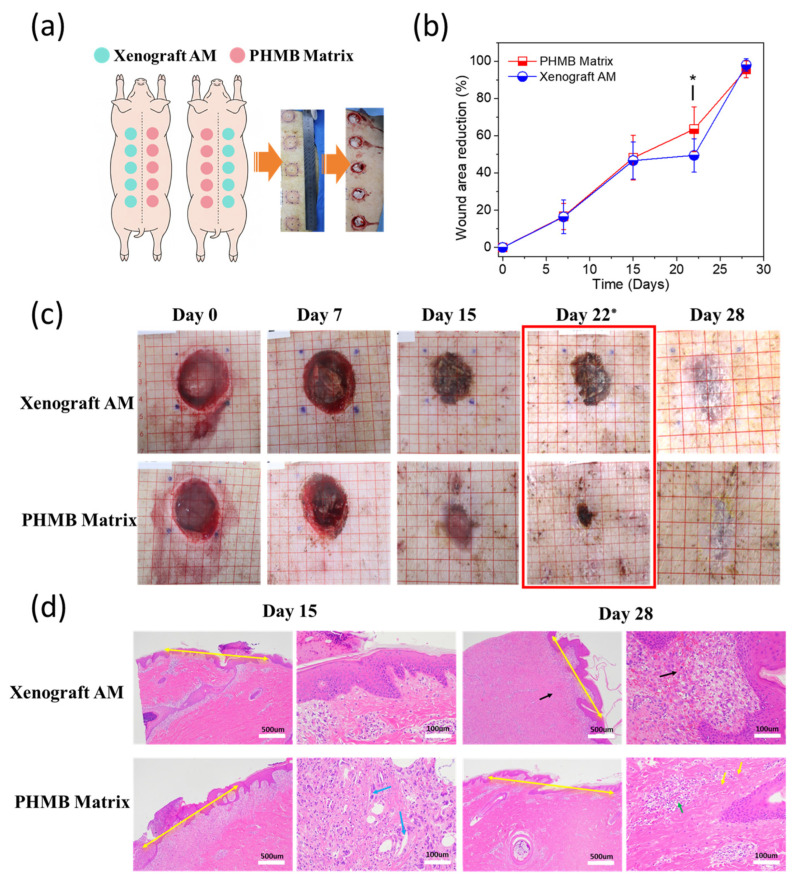
PHMB Matrix accelerates porcine wound healing. (**a**) Schematic of experimental design. Full-thickness wounds (3-cm diameter) were treated with PHMB Matrix or Xenograft AM. (**b**) Wound area reduction rate (mean ± SD). *n* = 40 initially (4 pigs, 10 wounds/pig); *n* = 20 from Day 15 onwards following scheduled sacrifice of 2 animals for histology. * *p* < 0.05 vs. Xenograft AM by Day 22 (*t*-test/Wilcoxon). (**c**) Representative macro photos. Background red grid = 1 cm × 1 cm. Images standardized to grid scale. Red box in (**c**) highlights the Day 22 time point corresponding to the onset of statistical significance. (**d**) Histology (H&E). Representative images on Days 15 and 28. Upper row: Xenograft AM; lower row: PHMB Matrix. For each group, left panels show low magnification (scale bar = 500 µm) and right panels show high magnification (scale bar = 100 µm). PHMB Matrix shows enhanced re-epithelialization and organized collagen deposition compared to Xenograft AM. Yellow double-headed arrows indicate wound gap width; black arrows indicate hemorrhage and loose tissue arrangement; cyan/blue arrows indicate macrophages; yellow single arrows indicate active fibroblast regions with organized collagen arrangement; green arrows indicate neovascularization.

To evaluate the repair efficacy of PHMB Matrix in a clinically relevant model, full-thickness excisional wounds (3-cm diameter) were bilaterally created on the dorsum of Bama minipigs and treated with either PHMB Matrix or Xenograft AM matrix (Figure 3a). A Bama minipig platform was selected due to the high-fidelity translational parallels between porcine and human integument, specifically regarding epidermal thickness, dermal-to-epidermal ratios, and sparse hair follicle distribution [38,39,68].

The PHMB Matrix was benchmarked against a commercially available, clinically broadly adopted Xenograft AM [69] with comparative performance metrics summarized in Table 1. Xenograft AM relies on surface-absorbed PHMB for rapid bioavailability, though its single-phase design lacks a long-term reservoir [70]. In contrast, the PHMB Matrix employs a dual-compartment strategy where degradation-coupled erosion of the fiber bulk sustains a persistent antibacterial shield throughout the tissue remodeling phase.

Poloxamer 188 (P188), used in clinically established constructs like PolyMem, functions as a surfactant that modulates the wound microenvironment [71,72,73,74]. In addition to facilitating continuous wound cleansing by reducing surface tension, P188 stabilizes cell membranes, preserving the viability of damaged cells and mitigating early inflammatory responses, including peri-wound edema [74]. By maintaining cellular integrity and controlling excessive inflammation, P188 supports a physiological environment conducive to re-epithelialization and granulation tissue formation [60,75].

Collectively, the PHMB Matrix comprises three functional components: (i) PHMB (2.5% *w*/*w*), embedded within the fiber matrix as a sustained-release antibacterial reservoir; (ii) a PLGA/PDO dual-fiber scaffold that provides structural support and governs degradation-coupled drug release kinetics, with synthetic polyester composition conferring inherent stability in protease-rich wound environments; and (iii) P188, which enhances surface wettability and stabilizes cell membranes at the wound interface. These components act in concert to provide sustained antibacterial protection, structural stability, and microenvironmental modulation throughout the wound healing cycle.

Planimetric analysis revealed numerically superior wound area reduction rates during the early inflammatory and proliferative phases, with both groups achieving 16.70% and 16.46% closure by Day 7, respectively, and 48.15% versus 46.69% by Day 15 (*p* > 0.05; Figure 3b). Notably, by Day 22, the PHMB Matrix group demonstrated significantly accelerated healing (63.53 ± 12.0%) compared to Xenograft AM (49.47% ± 9.0%, *p* < 0.05), suggesting enhanced matrix remodeling dynamics during the mid-remodeling phase. By Day 28, both treatments achieved near-complete epithelialization (95.64% vs. 98.01%, *p* > 0.05), confirming functional equivalence in the study at terminal assessment. This temporal pattern is clinically noteworthy because wound healing trajectory analyses have established that intermediate kinetic endpoints are robust predictors of overall therapeutic efficacy, independent of terminal closure rates [76,77]; accordingly, the statistically significant acceleration observed on Day 22 suggests a meaningful modulation of the reparative process that may reduce cumulative exposure to infection risk during the proliferative-to-remodeling transition.

Macroscopic observation further corroborated the quantitative findings. By Day 22 and Day 28, wounds treated with PHMB Matrix exhibited a notably smoother surface topology with minimal residual crusting, whereas the Xenograft AM-treated wounds displayed more prominent eschar formation and irregular wound margins (Figure 3c). By Day 28, the PHMB Matrix group showed a flat, well-integrated tissue surface closely resembling the surrounding intact skin, in contrast to the Xenograft AM group where residual scab and uneven surface contour remained visible.

Histopathological evaluation (ISO 10993-6:2016) yielded reactivity scores of 2.2 by Day 15 and 2.6 by Day 28, both classified as “minimal or no reaction,” with no tissue necrosis at any site. In Figure 3d, yellow double-headed arrows indicate wound gap width between wound margins. By Day 15, the PHMB Matrix group showed milder hemorrhage, active macrophage infiltration (Figure 3d, lower left panels, cyan arrows), and nascent neovascularization, indicating a well-coordinated early inflammatory-to-proliferative transition consistent with the accelerated wound closure observed by Day 22. By Day 28, the PHMB Matrix-treated wounds demonstrated organized collagen fiber bundles (Figure 3d, lower right panels, yellow arrows) and mature vasculature (Figure 3d, lower right panels, green arrows) with well-formed, continuous neo-epidermis and mature granulation tissue, whereas the Xenograft AM-treated wounds exhibited persistent hemorrhagic foci and looser tissue arrangement (Figure 3d, upper right panels, black arrows) with residual inflammatory infiltration. These findings collectively suggest that the PHMB Matrix not only provides comparable early-phase wound healing but confers a distinct advantage during the later remodeling phase, supported by earlier hemostatic control and neovascularization, resulting in improved macroscopic wound quality characterized by reduced scarring, less crusting, and improved tissue integration. All animals remained clinically healthy throughout the study with no device-related adverse events, and gross necropsy confirmed preserved organ integrity with no evidence of implant migration in draining lymph nodes.

### 3.3. Systemic Pharmacokinetics

**Figure 4:**
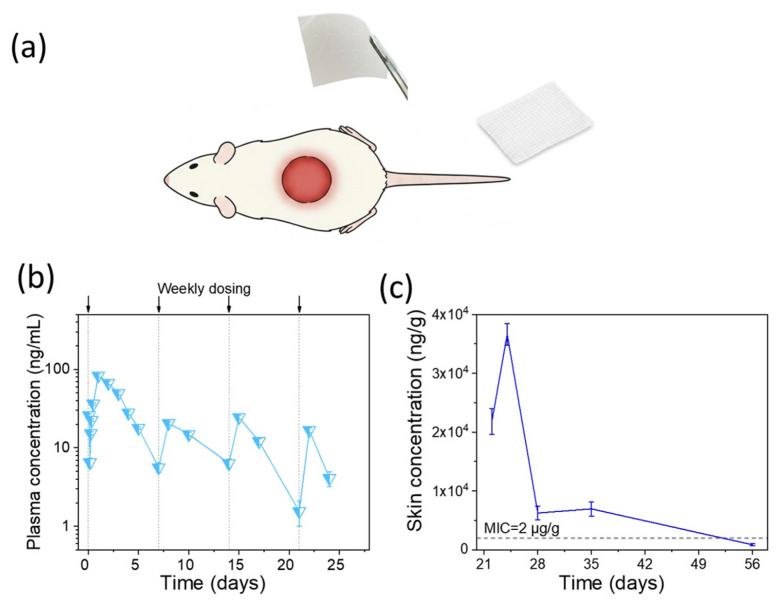
Pharmacokinetic profile of PHMB following repeated topical application of PHMB Matrix in a rat wound model. (**a**) Schematic illustration of the experimental procedure. A full-thickness skin wound was created on the dorsum of the rat, followed by topical application of PHMB Matrix (25 mg/g PHMB) over the wound bed. The matrix was replaced weekly. (**b**) Plasma concentration-time profile of PHMB over the 56-day study period (*n* = 8). Plasma PHMB concentrations remained in the low ng/mL range (predominantly 1–100 ng/mL) throughout the study period, with transient peaks observed following each application and rapid decline between doses, indicating minimal systemic absorption and rapid clearance. (**c**) PHMB concentration in wound-adjacent skin tissue (*n* = 6). The dashed line indicates the MIC reference value of 2 µg/g for common wound pathogens (*S. aureus*, MRSA, and *E. coli*) [24]. Note: During the active dosing phase, the majority of individual plasma concentrations fell below this threshold, and post-treatment samples (Day 25–56) were below the limit of detection (LOD). Data are expressed as Mean ± SEM. The theoretical dose was 1525 μg per rat.

To evaluate the absorption and wound-site retention of the PHMB Matrix, a full-thickness skin defect model in Sprague–Dawley (SD) rats (Figure 4a) was employed to systematically investigate the absorption and local tissue distribution profile of PHMB at a dosage of 25 mg/g [47,78,79,80]. Its absorption kinetics were characterized following four weekly topical applications (Days 0, 7, 14, and 21). By employing LC-MS/MS for the quantification of PHMB in plasma and wound site, we aimed to elucidate the correlation between the matrix’s in vitro release kinetics and its in vivo pharmacokinetic behavior. These findings provide critical preclinical evidence to support the clinical translation of this functional wound matrix.

As shown in Figure 4c, pharmacokinetic profiling in the rat wound model demonstrated that skin tissue PHMB concentrations rose gradually to a peak of approximately 35,000 ng/g by Day 25, rather than spiking immediately upon application, followed by a decline to a sustained plateau of approximately 5000 ng/g from Day 28 through Day 35 before returning to near-baseline by Day 56. Plasma concentrations remained below the LOD throughout Day 25 to 56 observation period (Figure 4b). As shown in Figure 4c, the residual tissue concentration of 5 μg/g exceeded published MIC values for common wound pathogens (*S. aureus*, *MRSA*, and *E. coli*) [24], confirming that therapeutically effective local drug concentrations were maintained throughout the sustained-release period of the matrix.

This seven-day tissue retention profile is consistent with, and mechanistically explained by, the controlled-release design of the PHMB Matrix. The loading concentration of 25 mg/g (2.5%), 2- to 25-fold higher than commercial PHMB dressings (0.1–1.1%), serves as a high-capacity reservoir rather than a bolus dose. Upon wound contact, progressive matrix hydration governs rate-limiting PHMB diffusion, accounting for the delayed concentration peak. The seven-day post-removal retention, attributed to electrostatic anchoring of cationic PHMB to anionic dermal matrix components, corroborates the in vitro release and degradation kinetics data showing sustained drug liberation over a seven-day cycle. Importantly, this seven-day antibacterial window is further validated by the AATCC antibacterial challenge testing, in which the PHMB Matrix maintained effective bactericidal activity under extreme microbial challenge conditions through the seven-day endpoint, establishing concordance across in vitro release, in vitro antibacterial challenge, and in vivo pharmacokinetic datasets. The rat model, with its inherently higher percutaneous permeability, represents a worst-case scenario for systemic exposure [49,81]; the porcine wound healing model, the recognized gold standard for clinical translation, demonstrated superior healing outcomes from Day 22 onward. Together, these complementary preclinical models support the PHMB Matrix as a once-weekly sustained-release antibacterial matrix achieving prolonged local efficacy with negligible systemic exposure.

## 4. Discussion

This study establishes a comprehensive, mechanistically grounded framework linking material architecture to clinical performance for a PHMB-functionalized fully absorbable synthetic wound matrix as an alternative to biologics. By tailoring the dynamic balance between matrix erosion and payload diffusion, the PHMB matrix achieves a critical equilibrium between sustained antibacterial efficacy, accelerated tissue repair, and safety.

**First, Sustained Antibacterial Efficacy Through Biphasic Release.** The dual-composite architecture (PLGA/PDO) that mimics ECM enables a biphasic release profile that aligns with the temporal antibacterial demands of wound healing [82]. The initial burst phase (~10% release within 1 h) delivers high localized PHMB concentrations during the critical early infection window (0–24 h post-injury), when the risk of bacterial colonization is maximal [25,83]. Subsequently, the sustained-release phase maintains therapeutic PHMB levels above the MIC99 for key pathogens throughout the inflammatory and proliferative phases (Days 1–21), achieving robust and durable broad-spectrum efficacy (LRV > 4.0; >99.99% reduction) against six clinically relevant pathogens with stability maintained after 15 months of real-time aging [57]. The residual ~45% PHMB molecularly entangled within the hydrophobic domains of the matrix is released gradually during polymer erosion, preventing the premature functional failure common in biologic products and extending antibacterial protection into the remodeling phase—a period where immunocompromised patients remain particularly vulnerable to opportunistic infections [8].

**Second, Coordinated Biodegradation Ensuring Uninterrupted Antibacterial Protection.** The matrix maintained structural integrity with negligible weight loss (<2%) during the first two weeks at 37 °C, providing essential mechanical support during the critical proliferative phase, followed by linear degradation reaching approximately 60% mass loss by Week 10. The observed “pH-weight loss lag”—rapid acidification preceding macroscopic mass loss—confirms that hydrolytic ester bond scission initiates PHMB liberation prior to structural disintegration, effectively eliminating the “sterile window gap” characteristic of surface-coated products and ensuring uninterrupted antibacterial protection throughout the 28-day wound healing cycle. Moreover, the lactic and glycolic acids generated during PLGA/PDO hydrolysis locally acidify the wound microenvironment, which has been demonstrated to potentiate innate antimicrobial defense and promote re-epithelialization [35,36]—thus conferring a secondary, scaffold-intrinsic therapeutic benefit beyond PHMB delivery alone.

**Third, Accelerated Repair in Porcine Wound Models.** In the full-thickness wound studies, the matrix achieved a wound area reduction of 63.53% ± 12.0% by Day 22, significantly exceeding that of Xenograft AM (49.47% ± 9.0%; *p* < 0.05). By Day 28, both treatments converged to near-complete epithelialization (95.64% vs. 98.01%, *p* > 0.05); at this timepoint, PHMB Matrix-treated wounds presented a flat, smooth surface closely resembling adjacent intact skin with minimal residual eschar, whereas Xenograft AM-treated wounds retained irregular contour and persistent crusting. Histological examination confirmed enhanced tissue remodeling characterized by a continuous neo-epidermis and organized dermal architecture, with no evidence of necrosis or adverse tissue reaction at either time point, demonstrating that the PHMB Matrix achieves enhanced wound healing outcomes while maintaining excellent biocompatibility. The superior repair capacity of the PHMB Matrix is therefore attributable to an accelerated mid-remodeling trajectory, driven by prolonged bioburden suppression and a stable healing microenvironment [84], rather than to a difference in terminal closure. Within the established wound healing trajectory framework, such intermediate kinetic acceleration is recognized as a valid and independent indicator of therapeutic efficacy [76,77], and the subsequent convergence on Day 28 confirms that this accelerated course shortens the infection-susceptible window without compromising final closure. Mechanistically, this advantage is directly linked to the biphasic release profile described above: sustained local PHMB concentrations above pathogen MICs throughout the 21-day infection-susceptible window—spanning the inflammatory and proliferative phases when the wound bed lacks an intact epithelial barrier [27,29,30]—ensure continuous antibacterial protection during the period of greatest vulnerability, thereby enabling unimpeded progression through the remodeling cascade.

While PHMB is a widely investigated antimicrobial agent with proven efficacy, its dose-dependent cytotoxicity represents a critical design consideration. Studies have documented that PHMB concentrations exceeding 0.04–0.1% can induce cytotoxic effects on keratinocytes, fibroblasts, and other wound-healing relevant cell types through membrane disruption mechanisms [85,86]. This necessitates careful balancing of antimicrobial potency with biocompatibility in PHMB-based wound care products. Our matrix addresses this challenge through controlled-release architecture that achieves biphasic PHMB delivery—initial burst for acute infection control followed by sustained low-level release— synchronized with biodegradation. This design maintains local PHMB concentrations within a therapeutic window sufficient for antimicrobial activity (>99.99% bacterial reduction) while avoiding cytotoxic peaks. The in vivo validation supports this strategy: histological examination revealed no evidence of cytotoxicity or tissue necrosis, and the PHMB Matrix achieved accelerated wound closure compared to the commercial control (63.53% vs. 49.47% closure on Day 22, *p* < 0.05). Pharmacokinetic analysis further confirmed localized drug action with negligible systemic exposure, demonstrating that the controlled-release system successfully mitigates cytotoxicity concerns while preserving therapeutic efficacy.

**Fourth, Favorable Safety Through Localized Enrichment and Efficient Metabolic Clearance.** A pharmacokinetic profile of localized enrichment with minimal systemic residue highlights the clinical safety of the PHMB Matrix. As the rat model typically yields a worst-case scenario for systemic exposure, the observed absence of PHMB in the circulation reinforces the matrix’s safety profile. Aligned with in vitro elution profiles and AATCC efficacy data, these findings demonstrate that the matrix functions as a sequestered antibacterial reservoir, providing potent local defense while precluding systemic risks [49,87].

In summary, the PHMB Matrix exemplifies a rational design strategy: tailoring composite fiber polymer architecture and degradation kinetics to achieve a release profile that mirrors the biological kinetics of infection risk and tissue repair in acute, complex, and chronic wounds. Critically, whereas prior PHMB-loaded electrospun platforms have been limited to monophasic burst release, non-resorbable scaffolds, or the absence of systemic safety data [31,32,33,34], the present PLGA/PDO matrix is, to our knowledge, the first to demonstrate concurrent biphasic release–biodegradation synchronization, accelerated wound repair in a translational large-animal model, and a confirmed favorable pharmacokinetic profile. This Structure–Release–Efficacy–Safety integration, validated across multi-scale models, provides a robust mechanistic foundation for clinical translation as a fully absorbable synthetic alternative to biologics.

**Limitations and Future Directions.** The findings of this study should be interpreted in light of several limitations. First, antibacterial efficacy was evaluated using the AATCC 100 planktonic assay, which demonstrated robust bactericidal performance (LRV > 4.0) against all six pathogens after 15 months of aging and 7-day SWF pre-depletion. This is corroborated by sustained local PHMB concentrations exceeding 10 μg/mL throughout the 21-day infection-susceptible window—substantially above planktonic MICs for *S. aureus* (0.5–2 μg/mL) and *P. aeruginosa* (2–8 μg/mL) [24]—and by infection-free healing in the porcine model. As a complementary extension, biofilm-specific studies employing Centers for Disease Control (CDC) biofilm reactor assays (American Society for Testing and Materials (ASTM) E2871) with confocal laser scanning microscopy (CLSM) imaging and biofilm-challenged porcine wound models are planned. Second, certain formulation parameters remain proprietary; the physicochemical, pharmacokinetic, and degradation data herein afford sufficient transparency for independent appraisal, and specifics may be disclosed under confidentiality upon request. Third, potential sponsor bias was mitigated through independent Contract Research Organization execution, randomization, blinding, and GLP compliance as detailed in Section 2.5. Fourth, the pH declines to ~3.0–3.5 observed during in vitro degradation occurred exclusively in non-buffered physiological saline; the buffered PBS condition maintained stable physiological pH (7.3–7.4) throughout the 10-week study. In vivo, intrinsic buffering mechanisms from serum proteins, bicarbonate, and tissue fluids are expected to attenuate local acidification, consistent with the absence of tissue necrosis and the minimal reactivity scores (2.2–2.6) observed in the porcine model. Ultimately, investigator-initiated studies and prospective clinical trials with larger cohorts are warranted to corroborate these findings [4,16,76,77,88].

## 5. Conclusions

In conclusion, this study establishes a fully absorbable PHMB-functionalized synthetic matrix as a high-performance alternative to biologic-derived materials for managing acute, complex, and chronic wounds. By leveraging a three-dimensional fibrous architecture that mimics ECM structure, the matrix achieves precise synchronization between its tuned biodegradation profile and biphasic PHMB release kinetics, ensuring a persistent antibacterial environment throughout the critical 21-day infection-susceptible window and beyond into the remodeling phase. In porcine wound models, the statistically significant acceleration by Day 22, followed by convergence by Day 28, demonstrates that the PHMB Matrix confers a kinetic advantage during the proliferative-to-remodeling transition—shortening the infection-susceptible window—while achieving equivalent final closure. Through quantitative demonstration of sustained antibacterial efficacy, accelerated tissue repair, and a favorable safety profile, these findings validate the PHMB Matrix as a safe and effective synthetic platform that directly addresses the inherent limitations of both biologic-derived matrices and existing PHMB-loaded electrospun systems in antibacterial durability, release control and enzymatic stability.

## Figures and Tables

**Table 1:** Comparison of PHMB Matrix and the commercial product Xenograft AM.

Property	PHMB Matrix	Xenograft AM
Structure	Synthetic Composite Fiber Matrix	Cross-linked Collagen Sheet
Composition	PLGA, PDO, Poloxamer 188, PHMB	Type I Collagen, PHMB
Antibacterial Agent	PHMB	PHMB
Concentration	2.5% (*w*/*w*)	0.1% (*w*/*w*)
Integration Method	Embedded in Fiber	Interfacial Coating
Release Behavior	Biphasic controlled, sustained	Monophasic, rapid depletion

## Data Availability

Raw data are available for independent verification upon reasonable request.
